# Supplementary material for: Bioavailability and Metabolic Fate of (Poly)phenols from Hull-Less Purple Whole-Grain Barley in Humans
Source: Nutrients. 2025 Sep 28;17(19):3086. doi: 10.3390/nu17193086 (PMC12526210; doi:10.3390/nu17193086)
Supplement: Supplementary file 1 [file nutrients-17-03086-s001.zip › Supplemental Table S1_Cortijo-Alfonso_Nutrients.pdf]

Supplemental Table S1. Nutritional composition per portion of the testing meal WGB biscuits (140 g).

	**Whole grain barley (WGB) biscuits (140 g)**
Energy (Kcal)	617
Energy (KJ)	2584
Fats (g)	14,7
Carbohydrates (g)	104
Fiber (g)	7,93
Soluble	4,89
Insoluble	3,04
Proteins (g)	13,6
Amino acids	
Phenylalanine (mg)	710
Tyrosine (mg)	310
