# Supplementary material for: Bioavailability and Metabolic Fate of (Poly)phenols from Hull-Less Purple Whole-Grain Barley in Humans
Source: Nutrients. 2025 Sep 28;17(19):3086. doi: 10.3390/nu17193086 (PMC12526210; doi:10.3390/nu17193086)
Supplement: Supplementary file 1 [file nutrients-17-03086-s001.zip › Supplemental Table S2_Cortijo-Alfonso_Nutrients.pdf]

Supplemental Table S2. Selected reaction monitoring (SRM) conditions used for the quantification of the phenolic compounds in WGB biscuits and the generated metabolites after its acute intake.

(Poly)phenolic compounds	MW (g/mol)	Ionization mode (ESI)	SRM Quantification		
			Transition	Cone Voltage (V) / Collision energy (eV)	Phenolic in which has been (tentatively) quantified
Cyanidin-3-O-glucoside	448	Positive	449 > 287	40 / 20	Cyanidin-3-*O*-glucoside
Cyanidin-3-(3′′-malonylglucoside)	534	Positive	535 > 287	40 / 15	Cyanidin-3-*O*-glucoside
Cyanidin-3-(6′′-malonylglucoside)	534	Positive	535 > 287	40 / 15	Cyanidin-3-*O*-glucoside
Cyanidin-3-*O*-(3′′,6′′)-dimalonylglucoside	620	Positive	621 > 287	40 / 20	Cyanidin-3-*O*-glucoside
Peonidin-3-*O*-Glucoside	462	Positive	463 > 301	40 / 20	Cyanidin-3-*O*-glucoside
Peonidin-3-*O*-6′′-O-malonylglucoside	548	Positive	549 > 301	40 / 20	Cyanidin-3-*O*-glucoside
Peonidin-3-*O*-(3′′,6′′)-dimalonylglucoside	634	Positive	635 > 301	40 / 20	Cyanidin-3-*O*-glucoside
Peonidin-3-*O*-Glucuronide	476	Positive	477 > 301	40 / 15	Cyanidin-3-*O*-glucoside
Pelargonidin-3-*O*-Glucoside	432	Positive	433 > 271	40 / 20	Cyanidin-3-*O*-glucoside
Pelargonidin-3-*O*-(3′′-malonylglucoside)	518	Positive	519 > 271	40 / 20	Cyanidin-3-*O*-glucoside
Pelargonidin-3-*O*-(6′′-malonylglucoside)	518	Positive	519 > 271	40 / 20	Cyanidin-3-*O*-glucoside
Delphinidin-3-*O*-Glucoside	454	Positive	465 > 303	40 / 20	Cyanidin-3-*O*-glucoside
4-hydroxybenzoic acid	138	Negative	137 > 93	30 / 15	4-hydroxybenzoic acid
Hydroxybenzoic acid	138	Negative	137 > 93	30 / 15	4-hydroxybenzoic acid
Hydroxybenzoic acid-*O*-sulphate	218	Negative	217 > 137	35 / 15	4-hydroxybenzoic acid
3,4-dihydroxybenzoic acid	154	Negative	153 > 109	45 / 15	3,4-dihydroxybenzoic acid
3,4-dihydroxybenzoic acid-O-sulphate	234	Negative	233 > 153	35 / 15	3,4-dihydroxybenzoic acid
4-hydroxy-3-methoxybenzoic acid	168	Negative	167 > 152	30 / 10	4-hydroxy-3-methoxybenzoic acid
4-hydroxy-3-methoxybenzoic acid-*O*-sulphate	248	Negative	247 > 167	20 / 15	4-hydroxy-3-methoxybenzoic acid
4-hydroxy-3-methoxybenzoic acid-*O*-glucuronide	344	Negative	343 > 167	40 / 10	4-hydroxy-3-methoxybenzoic acid
4-hydroxy-3-methoxybenzoic acid-*O*-glycine	225	Negative	224 > 123	40 / 10	4-hydroxy-3-methoxybenzoic acid
4-Hydroxy-3,5-dimethoxybenzoic acid	198	Negative	197 > 182	30 / 10	4-hydroxy-3,5-dimethoxybenzoic acid
4-Hydroxy-3,5-dimethoxybenzoic acid-*O*-sulphate	278	Negative	277 > 197	40 / 20	4-hydroxy-3,5-dimethoxybenzoic acid
Cinnamic acid	148	Negative	147 > 103	20 / 10	4′-hydroxycinnamic acid
4′-Hydroxycinnamic acid	164	Negative	163 > 119	35 / 15	4′-hydroxycinnamic acid
4′-Hydroxycinnamic acid-*O*-sulphate	244	Negative	243 > 163	35 / 15	4′-hydroxycinnamic acid

3′,4′-Dihydroxycinnamic acid	180	Negative	179 > 135	35 / 15	3,4-dihydroxycinnamic acid
3′,4′-Dihydroxycinnamic acid-*O*-sulphate	260	Negative	259 > 179	35 / 15	3,4-dihydroxycinnamic acid
4′-Hydroxy-3′-methoxycinnamic acid	194	Negative	193 > 134	30 / 15	4′-Hydroxy-3′-methoxycinnamic acid
3′-Hydroxy-4′-methoxycinnamic acid	194	Negative	193 > 134	30 / 15	4′-Hydroxy-3′-methoxycinnamic acid
4′-Hydroxy-3′-methoxycinnamic acid-*O*-sulphate	274	Negative	273 > 193	40 / 10	4′-Hydroxy-3′-methoxycinnamic acid
3′-Hydroxy-4′-methoxycinnamic acid-*O*-sulphate	274	Negative	273 > 193	40 / 10	4′-Hydroxy-3′-methoxycinnamic acid
4′-Hydroxy-3′-methoxycinnamic acid-*O*-glucuronide	370	Negative	369 > 193	40 / 20	4′-Hydroxy-3′-methoxycinnamic acid
3′-Hydroxy-4′-methoxycinnamic acid-*O*-glucuronide	370	Negative	369 > 193	40 / 20	4′-Hydroxy-3′-methoxycinnamic acid
4′-Hydroxy-3′-methoxycinnamic acid-*O*-glycine	249	Negative	250 > 149	40 / 15	4′-Hydroxy-3′-methoxycinnamic acid
3′-Hydroxy-4′-methoxycinnamic acid-*O*-glycine	249	Negative	250 > 206	40 / 10	4′-Hydroxy-3′-methoxycinnamic acid
4′-Hydroxy-3,5-dimethoxycinnamic acid	224	Negative	223 > 179	35 / 15	4-Hydroxy-3,5-dimethoxycinnamic acid
3-(4-Hydroxy-3,5-dimethoxyphenyl)propanoic acid	226	Negative	225 > 181	40 / 15	4-Hydroxy-3,5-dimethoxycinnamic acid
3-(4-Hydroxy-3,5-dimethoxyphenyl)propanoic acid-O-sulphate (I)	306	Negative	305 > 225	40 / 15	4-Hydroxy-3,5-dimethoxycinnamic acid
3-(4-Hydroxy-3,5-dimethoxyphenyl)propanoic acid-O-sulphate (II)	306	Negative	305 > 225	40 / 15	4-Hydroxy-3,5-dimethoxycinnamic acid
4′-Hydroxy-3,5-dimethoxycinnamic acid-*O*-glucoside	386	Negative	385 > 223	40 / 15	4-Hydroxy-3,5-dimethoxycinnamic acid
3-(4′-Hydroxy-3′-methoxyphenyl)propanoic acid	196	Negative	195 > 135	35 / 15	3-(4′-Hydroxy-3′-methoxy-phenyl)propanoic acid
3-(4′-Hydroxy-3′-methoxyphenyl)propanoic acid-*O*-sulphate	276	Negative	275 > 195	35 / 15	3-(4′-Hydroxy-3′-methoxy-phenyl)propanoic acid
3-(3′-Hydroxy-4′-methoxyphenyl)propanoic acid-*O*-sulphate	276	Negative	275 > 195	35 / 15	3-(4′-Hydroxy-3′-methoxy-phenyl)propanoic acid
3-(4′-Hydroxy-3′-methoxyphenyl)propanoic acid-*O*-glucuronide	372	Negative	371 > 195	35 / 15	3-(4′-Hydroxy-3′-methoxy-phenyl)propanoic acid
3-(3′-Hydroxy-4′-methoxyphenyl)propanoic acid-*O*-glucuronide	372	Negative	371 > 195	35 / 15	3-(4′-Hydroxy-3′-methoxy-phenyl)propanoic acid
Diferulic acid	386	Negative	385 > 341	40 / 15	4′-Hydroxy-3′-methoxycinnamic acid
Diferulic acid (decarboxylated form)	342	Negative	341 > 282	40 / 15	4′-Hydroxy-3′-methoxycinnamic acid
Triferulic acid	578	Negative	577 > 355	50 / 20	4′-Hydroxy-3′-methoxycinnamic acid
Catechin	290	Negative	289 > 245	45 / 15	Catechin
Catechin-*O*-glucoside	452	Negative	451 > 289	45 / 15	Catechin
Procyanidin B3	578	Negative	577 > 289	45 / 20	Catechin
Gallocatechin catechin or prodelphinidin B4	594	Negative	593 > 289	45 / 20	Catechin

Catechin-*O*-sulphate	370	Negative	369 > 289	40 / 20	Catechin
Methyl catechin-*O*-sulphate	384	Negative	383 > 289	40 / 20	Catechin
Methyl epicatechin-*O*-glucuronide	480	Negative	479 > 303	40 / 25	Catechin
5-(4′-hydroxyphenyl)-g-valerolactone-*O*-sulphate	272	Negative	271 > 191	40 / 20	Catechin
5-(4′-hydroxyphenyl)-g-valerolactone-*O*-glucuronide (I)	368	Negative	367 > 191	40 / 20	Catechin
5-(4′-hydroxyphenyl)-g-valerolactone-*O*-glucuronide (II)	368	Negative	367 > 191	40 / 20	Catechin
5-(3′,4′-dihydroxyphenyl)-g-valerolactone-*O*-sulphate (I)	288	Negative	287 > 207	40 / 15	Catechin
5-(3′,4′-dihydroxyphenyl)-g-valerolactone-*O*-sulphate (II)	288	Negative	287 > 207	40 / 15	Catechin
5-(3′,4′-dihydroxyphenyl)-g-valerolactone-*O*-glucuronide (I)	384	Negative	383 > 207	40 / 20	Catechin
5-(3′,4′-dihydroxyphenyl)-g-valerolactone-*O*-glucuronide (II)	384	Negative	387 > 207	40 / 20	Catechin
5-(3′,4′-dihydroxyphenyl)-g-valerolactone-*O*-glucuronide (II)	384	Negative	387 > 207	40 / 20	Catechin
5-(3′,4′-dihydroxyphenyl)-g-valerolactone-*O*-sulphate-O-glucuronide	464	Negative	463 > 287	40 / 20	Catechin
Apigenin-*O*-glucoside	432	Negative	431 > 269	40 / 25	Luteolin
Apigenin-6-*C*-arabinoside-8-*C*-glucoside	564	Negative	563 > 353	60 / 25	Luteolin
Isovitexin-*C*-glucoside	594	Negative	593 > 431	60 / 20	Luteolin
Isovitexin-*C*-rutinoside	740	Negative	739 > 431	60 / 20	Luteolin
Isoscoparin-*C*-glucoside	624	Negative	623 > 341	60 / 30	Luteolin
Isoscoparin-*C*-rutinoside	770	Negative	769 > 461	60 / 20	Luteolin
Luteolin	286	Negative	285 > 133	40 / 30	Luteolin
Chrysoeriol	300	Positive	301 > 286	40 / 30	Methyl Luteolin
Luteolin-*O*-glucoside	448	Negative	447 > 285	40 / 20	Luteolin
Luteolin-7-*O*-glucuronide	462	Positive	463 > 287	40 / 20	Luteolin-7-*O*-glucuronide
Chrysoeriol-*O*-glucoside	462	Negative	461 > 299	40 / 20	Luteolin
Chrysoeriol-*O*-glucuronide	476	Positive	477 > 301	40 / 20	Luteolin-7-*O*-glucuronide
Eriodictyol-*O*-sulphate	368	Negative	367 > 287	40 / 20	Luteolin
Eriodictyol-*O*-glucuronide	464	Negative	463 > 287	40 / 20	Luteolin
Hippuric acid	179	Negative	178 > 134	40 / 10	4-hydroxybenzoic acid
Hydroxyhippuric acid	195	Negative	194 > 100	40 / 10	4-hydroxybenzoic acid
4-Hydroxyphenylacetic acid	152	Negative	151 > 107	20 / 10	4-hydroxybenzoic acid
2-Hydroxyphenylacetic acid	152	Negative	151 > 107	20 / 10	4-hydroxybenzoic acid
Hydroxyphenylacetic acid-*O*-sulphate	232	Negative	231 > 151	20 / 15	3-(4′-Hydroxyphenyl)propanoic acid
Hydroxyphenylacetic acid-*O*-glucuronide	328	Negative	327 > 151	20 / 10	3-(4′-Hydroxyphenyl)propanoic acid
Dihydroxyphenylacetic acid-*O*-sulphate	248	Negative	247 > 167	40 / 10	3-(3′,4′-Dihydroxyphenyl)propanoic acid

Dihydroxyphenylacetic acid-*O*-glucuronide	344	Negative	343 > 167	40 / 10	3-(3′,4′-Dihydroxyphenyl)propanoic acid
3-(4′-Hydroxyphenyl)propanoic acid	166	Negative	165 > 121	20 / 10	3-(4′-Hydroxyphenyl)propanoic acid
3-(4′-Hydroxyphenyl)propanoic acid-*O*-sulphate (I)	246	Negative	245 > 165	35 / 15	3-(4′-Hydroxyphenyl)propanoic acid
3-(4′-Hydroxyphenyl)propanoic acid-*O*-sulphate (II)	246	Negative	245 > 165	35 / 15	3-(4′-Hydroxyphenyl)propanoic acid
3-(4′-Hydroxyphenyl)propanoic acid-*O*-glucuronide	342	Negative	341 > 165	40 / 25	3-(4′-Hydroxyphenyl)propanoic acid
3-(3′,4′-Dihydroxyphenyl)propanoic acid	182	Negative	181 > 137	40 / 15	3-(3′,4′-Dihydroxyphenyl)propanoic acid
3-(3′,4′-Dihydroxyphenyl)propanoic acid-*O*-sulphate (I)	262	Negative	261 > 181	40 / 15	3-(3′,4′-Dihydroxyphenyl)propanoic acid
3-(3′,4′-Dihydroxyphenyl)propanoic acid-*O*-sulphate (II)	262	Negative	261 > 181	40 / 15	3-(3′,4′-Dihydroxyphenyl)propanoic acid
3-(3′,4′-Dihydroxyphenyl)propanoic acid-*O*-glucuronide	358	Negative	357 > 181	40 / 15	3-(3′,4′-Dihydroxyphenyl)propanoic acid
1,2-Dihydroxybenzene	110	Negative	108.9 > 90.9	20 / 15	1,2-Dihydroxybenzene
1,2-Dihydroxybenzene-*O*-sulphate (I)	190	Negative	189 > 109	20 / 15	1,2-Dihydroxybenzene
1,2-Dihydroxybenzene-*O*-sulphate (II)	190	Negative	189 > 109	20 / 15	1,2-Dihydroxybenzene
Methoxy-hydroxybenzene-*O*-sulphate (I)	204	Negative	203 > 123	20 / 15	1,2-Dihydroxybenzene
Methoxy-hydroxybenzene-*O*-sulphate (II)	204	Negative	203 > 123	20 / 15	1,2-Dihydroxybenzene
1,2-Dihydroxybenzene-*O*-glucuronide (I)	300	Negative	299 > 123	40 / 15	1,2-Dihydroxybenzene
1,2-Dihydroxybenzene-*O*-glucuronide (II)	300	Negative	299 > 123	40 / 15	1,2-Dihydroxybenzene
1,2,3-Trihydroxybenzene-*O*-sulphate (I)	206	Negative	205 > 125	20 / 15	1,2-Dihydroxybenzene
1,2,3-Trihydroxybenzene-*O*-sulphate (II)	206	Negative	205 > 125	20 / 15	1,2-Dihydroxybenzene
1,3,5-Trihydroxybenzene-*O*-sulphate (I)	206	Negative	205 > 125	20 / 15	1,2-Dihydroxybenzene
1,3,5-Trihydroxybenzene-*O*-sulphate (II)	206	Negative	205 > 125	20 / 15	1,2-Dihydroxybenzene
3-Methoxy-1,5-dihydroxybenzene-*O*-sulphate (I)	220	Negative	219 > 139	20 / 15	1,2-Dihydroxybenzene
3-Methoxy-1,5-dihydroxybenzene-*O*-sulphate (II)	220	Negative	219 > 139	20 / 15	1,2-Dihydroxybenzene
3-Methoxy-1,5-dihydroxybenzene-*O*-glucuronide	316	Negative	315 > 139	20 / 20	1,2-Dihydroxybenzene

MW: Molecular weight
