# Supplementary material for: Bioavailability and Metabolic Fate of (Poly)phenols from Hull-Less Purple Whole-Grain Barley in Humans
Source: Nutrients. 2025 Sep 28;17(19):3086. doi: 10.3390/nu17193086 (PMC12526210; doi:10.3390/nu17193086)
Supplement: Supplementary file 1 [file nutrients-17-03086-s001.zip › Supplemental Table S3_Cortijo-Alfonso_Nutrients.pdf]

Supplemental Table S3. Urine Concentration (µmols) expressed as medium value ± standard error of mean (SEM) excreted at different time intervals after an acute intake of purple whole grain barley (WGB) for the eleven volunteers. Number of replicates was two.

(Poly)phenolic metabolites	0 h (Basal)	0-2 h	2-4 h	4-8 h	8-24 h	24-48 h
**Anthocyanins ***						
Peonidin-3-*O*-glucoside	0.55 ± 0.15 [a]	1.91 ± 0.91 [a,b]	5.64 ± 2.18 [b]	1.92 ± 1.06 [a,b]	2.63 ± 1.38 [a,b]	1.55 ± 1.55 [a]
Peonidin-3-*O*-glucuronide	0.13 ± 0.09 [a]	0.39 ± 0.18 [a]	2.52 ± 0.79 [a,b]	3.96 ± 2.84 [b]	0.34 ± 0.23 [a]	0.12 ± 0.12 [a]
Cyanidin-3-*O*-(3''-*O*-malonyl)glucoside	n.d. [a]	0.06 ± 0.04 [a]	0.54 ± 0.21 [a]	2.76 ± 1.02 [b]	2.03 ± 0.52 [b]	n.d. [a]
Cyanidin-3-*O*-(6''-*O*-malonyl)glucoside	n.d. [a]	0.15 ± 0.09 [a]	1.09 ± 0.31 [a,b]	3.42 ± 1.70 [b]	3.03 ± 1.10 [b]	n.d. [a]
**Flavan-3-ols**						
Catechin-*O*-sulphate	n.d. [a]	5.70 ± 3.59 [b,c]	8.48 ± 1.67 [c]	5.25 ± 1.02 [b,c]	2.93 ± 0.90 [a,b]	1.46 ± 1.46 [a,b]
Methyl catechin-*O*-sulphate	n.d. [a]	0.76 ± 1.04 [a,b]	2.33 ± 1.27 [c]	2.03 ± 0.94 [b,c]	3.66 ± 2.54 [d]	0.55 ± 1.82 [a]
Methyl epicatechin-*O*-glucuronide	n.d. [a]	0.15 ± 0.09 [a,b]	0.47 ± 0.06 [b,c]	0.43 ± 0.12 [b]	0.85 ± 0.32 [c]	0.00 ± 0.00 [a]
Hydroxyphenyl-γ-valerolactone-*O*-sulphate	n.d. [a]	n.d. [a]	n.d. [a]	4.25 ± 3.63 [a]	68.0 ± 44.0 [b]	13.2 ± 4.98 [a]
Hydroxyphenyl-γ-valerolactone-*O*-glucuronide (I)	n.d. [a]	n.d. [a]	n.d. [a]	0.44 ± 0.26 [a]	2.09 ± 0.67 [b]	0.76 ± 0,76 [a]
Hydroxyphenyl-γ-valerolactone-*O*-glucuronide (II)	n.d. [a]	n.d. [a]	n.d. [a]	0.38 ± 0.37 [a]	7.76 ± 6.00 [b]	1.06 ± 0.49 [a,b]
Dihydroxyphenyl-γ-valerolactone-*O*-sulphate (I)	0.46 ± 0.12 [a]	0.40 ± 0.17 [a]	1.14 ± 0.30 [a,b]	21.5 ± 14.8 [a,b]	232 ± 58.8 [c]	87.2 ± 43.5 [b]
Dihydroxyphenyl-γ-valerolactone-*O*-sulphate (II)	3.99 ± 1.26 [a]	0.48 ± 0.37 [a]	60.8 ± 32.0 [a]	723 ± 347 [a]	3947 ± 506 [b]	578 ± 264 [a]
Dihydroxyphenyl-γ-valerolactone-*O*-glucuronide (I)	n.d. [a]	n.d. [a]	0.28 ± 0.16 [a,b]	8.21 ± 4.33 [b]	39.3 ± 5.03 [c]	4.86 ± 1.92 [a,b]
Dihydroxyphenyl-γ-valerolactone-*O*-glucuronide (II)	n.d. [a]	n.d. [a]	1.18 ± 0.74 a	19.2 ± 9.01 [a]	124 ± 31.4 [b]	12.9 ± 5.21 [a]
Dihydroxyphenyl-γ-valerolactone-*O*-sulphate glucuronide	n.d. [a]	n.d. [a]	0.13 ± 0.09 [a]	5.53 ± 3.46 [a]	45.5 ± 7.43 [b]	5.86 ± 3.11 [a]
**Flavones**						
Luteolin	n.d.	n.d. [a]	0.50 ± 0.20 [a,b]	1.06 ± 0.52 [c]	1.09 ± 0.50 [b,c]	n.d. [a]
Luteolin-*O*-sulphate	0.36 ± 0.11 [a]	1.30 ± 0.42 [a,b]	1.83 ± 0.57 [a,b]	2.82 ± 0.87 [b]	2.39 ± 0.59 [b]	1.22 ± 0.70 [a,b]
Luteolin-*O*-glucuronide	1.13 ± 0.23 [a]	1.70 ± 0.67 [a,b]	3.90 ±1.27 [a,b]	16.2 ± 5.41 [b,c]	22.7 ± 11.5 [c]	1.38 ± 1.10 [a,b]
Chrysoeriol	n.d. [a]	0.39 ± 0.16 [a]	0.82 ± 0.24 [a]	17.3 ± 13.6 [b]	6.64 ± 2.61 [a,b]	0.80 ± 0,35 [a]
Chrysoeriol-*O*-sulphate	0.17 ± 0.04 [a]	1.00 ± 0.28 [a]	2.41 ± 0.74 [a,b]	4.75 ± 0.95 [b,c]	5,69 ± 1.59 [c]	0.44 ± 0.40 [a]
Chrysoeriol-*O*-glucuronide	0.74 ± 0.22 [a]	79.7 ± 20.0 [a]	274 ± 50.3 [a]	753 ± 208 [b]	1055 ± 343 [b]	51.0 ± 21.9 [a]
Eriodictyol-*O*-sulphate	n.d. [a]	0.22 ± 0.12 [a]	0.16 ± 0.12 [a]	0.72 ± 0.24 [a]	1.94 ± 0.61 [b]	0.59 ± 0.40 [a]
Eriodictyol-*O*-glucuronide	0.09 ± 0.03 [a]	0.06 ± 0.04 [a]	0.10 ± 0.07 [a]	0.05 ± 0.05 [a]	0.85 ± 0.05 [b]	n.d. [a]
**Phenolic acids**						
Hydroxybenzoic acids						
4-hydroxybenzoic acid	7.04 ± 1.19 [a]	8.82 ± 3.92 [a]	4.95 ± 1.47 [a]	6.02 ± 2.88 [a]	16.9 ± 7.33 [a]	61.8 ± 29.5 [b]
Hydroxybenzoic acid	1.39 ± 0.58 [a,b]	0.30 ± 0.20 [a]	0.39 ± 0.27 [a]	0.97 ± 0.53 [a]	11.8 ± 7.14 [c]	11.5 ± 5.40 [b,c]
Hydroxybenzoic acid-*O*-sulphate	40.3 ± 5.69 [a]	53.1 ± 17.9 [a]	37.8 ± 23.1 [a]	37.2 ± 16.7 [a]	27.10 ± 19.08 [a]	261.6 ± 131 [b]

Hippuric acid	205 ± 26.9 [a]	387 ± 157 [a]	227 ± 71.0 [a]	324 ± 160 [a]	793 ± 394 [a]	2726 ± 1066 [b]
Hydroxyhippuric acid	19.7 ± 3.17 [a]	33.3 ± 5.22 [a]	21.8 ± 6.73 [a]	17.0 ± 7.16 [a]	48.8 ± 17.0 [a]	118 ± 52.1 [b]
3,4-dihydroxybenzoic acid	3.14 ± 6.66 [a]	13.5 ± 4.40 [a,b]	38.3 ± 6.15 [c,d]	27.4 ± 6.14 [b,c,d]	43.6 ± 11.3 [d]	22.2 ± 5.74 [b,c]
3,4-dihydroxybenzoic acid-*O*-sulphate	15.1 ± 3.87 [a]	22.4 ± 7.14 [a]	54.8 ± 12.1 [a,b]	53.8 ± 18.7 [a,b]	141 ± 49.4 [b]	142 ± 75.6 [b]
3,5-dimethoxy-4-hydroxybenzoic acid	0.24 ± 0.09 [a]	0.35 ± 0.17 [a]	0.44 ± 0.15 [a,b]	0.29 ± 0.12 [a]	1.76 ± 1.04 [b]	1.06 ± 0.49 [a,b]
3,5-dimethoxy-4-hydroxybenzoic acid-*O*-sulphate	0.68 ± 0.33 [a]	0.32 ± 0.18 [a]	0.30 ± 0.18 [a]	0.69 ± 0.30 [a]	3.67 ± 1.52 [b]	1.67 ± 0.76 [a,b]
4-hydroxy-3-methoxybenzoic acid	0.56 ± 0.20 [a]	1.40 ± 0.43 [a]	2.48 ± 0.56 [a]	3.24 ± 0.95 [a]	8.57 ± 2.41 [a,b]	13.8 ± 7.49 [b]
4-hydroxy-3-methoxybenzoic acid-*O*-sulphate	12.5 ± 2.92 [a]	23.6 ± 9.00 [a,b]	40.6 ± 10.4 [a,b]	30.2 ± 11.3 [a,b]	198 ± 125 [a,b]	312 ± 218 [b]
4-hydroxy-3-methoxybenzoic acid-*O*-glucuronide	0.26 ± 0.08 [a]	0.29 ± 0.11 [a]	1.01 ± 0.23 [a]	1.13 ± 0.41 [a]	4.48 ± 1.56 [a,b]	5.79 ± 3.51 [b]
4-hydroxy-3-methoxybenzoic acid-*O*-glycine	0.97 ± 0.23 [a]	3.22 ± 1.23 [a,b]	4.63 ± 0.79 [a,b]	4.35 ± 1.87 [a,b]	20.6 ± 12.0 [b]	18.4 ± 11.7 [a,b]
Hydroxycinnamic acids						
4’-hydroxycinnamic acid	0.14 ± 0.07 [a]	0.15 ± 0.08 [a,b]	0.23 ± 0.12 [a,b]	0.20 ± 0.11 [a,b]	0.89 ± 0.62 [b]	n.d. [a]
4’-hydroxycinnamic acid-*O*-sulphate	0.27 ± 0.17 [a]	3.56 ± 1.76 [a]	2.11 ± 0.52 [a]	1.61 ± 0.64 [a]	2.81 ± 1.48 [a]	9.13 ± 3.39 [b]
3’,4’-dihydroxycinnamic acid	3.70 ± 0.53 [a]	11.5 ± 4.52 [a]	29.9 ± 7.20 [a,b]	32.5 ± 8.10 [a,b]	46.6 ± 17.3 [a,b]	118 ± 89.3 [b]
3’,4’-dihydroxycinnamic acid-*O*-sulphate	3.10 ± 0.76 [a]	7.56 ± 4.78 [a]	18.3 ± 5.00 [a,b]	13.7 ± 4.97 [a,b]	24.5 ± 9.96 [a,b]	93.2 ± 70.3 [b]
4’-hydroxy-3’-methoxycinnamic acid	0.08 ± 0.03 [a]	0.25 ± 0.18 [a]	0.92 ± 0.25 [a]	2.16 ± 0.64 [a]	6.42 ± 1.86 [b]	1.10 ± 0.55 [a]
3’-hydroxy-4’-methoxycinnamic acid	0.04 ± 0.02 [a]	0.12 ± 0.07 [a]	0.29 ± 0.15 [a]	0.18 ± 0.16 [a]	0.95 ± 0.41 [b]	0.12 ± 0.12 [a]
3-(4’-hydroxy-3’-methoxyphenyl)propanoic acid	2.53 ± 0.54 [a]	23.1 ± 13.3 [a,b]	21.6 ± 6.54 [a,b]	103 ± 29.4 [b]	364 ± 69.5 [a]	84.5 ± 26.2 [a,b]
4’-hydroxy-3’-methoxycinnamic acid-*O*-glycine	5.51 ±1.57 [a]	13.4 ± 5.34 [a]	16.4 ± 5.20 [a]	74.4 ± 30.7 [a]	317 ± 80.8 [b]	36.9 ± 9.97 [a]
3’-hydroxy-4’-methoxycinnamic acid-*O*-glycine	2.03 ± 3.22 [a]	7.00 ± 1.52 [a]	3.15 ± 1.97 [a]	29.2 ± 1.23 [a]	111 ± 3.68 [b]	9.95 ± 7.75 [a]
4’-hydroxy-3’-methoxycinnamic acid-*O*-sulphate	11.9 ± 3.00 [a]	24.08 ± 1.58 [a]	57.0 ± 1.88 [a]	48.1 ± 6.14 [a,b]	107 ± 17.1 [b]	61.9 ± 13.3 [a,b]
3’-hydroxy-4’-methoxycinnamic acid-*O*-sulphate	0.85 ± 0.28 [a]	1.99 ± 0.75 [a,b]	3.17 ± 0.90 [a,b,c]	1.87 ± 0.97 [a]	6.41 ± 1.82 [c]	5.32 ± 1.73 [b,c]
3-(4’-hydroxy-3’-methoxyphenyl)propanoic acid	20.1 ± 7.47 [a]	54.8 ± 21.6 [a]	24.1 ± 10.2 [a]	212 ± 121 [a]	795 ± 117 [b]	116 ± 48.7 [a]
3-(3’-hydroxy-4’-methoxyphenyl)propanoic acid	n.d.[a]	n.d. [a]	n.d. [a]	n.d. [a]	3.95 ± 2.66 [a,b]	13.1 ± 10.1 [b]
4’-hydroxy-3’-methoxycinnamic acid-*O*-glucuronide	0.68 ± 0.23 [a]	0.85 ± 0.43 [a,b]	3.30 ± 0.66 [b]	3.33 ± 0.89 [b]	4.64 ± 0.95 [b]	3.01 ± 1.11 [a,b]
3’-hydroxy-4’-methoxycinnamic acid-*O*-glucuronide	0.06 ± 0.02 [a]	0.06 ± 0.04 [a]	0.11 ± 0.07 [a]	0.07 ± 0.06 [a]	0.28 ± 0.19 [a,b]	0.56 ± 0.30 [b]
3-(4’-hydroxy-3’-methoxyphenyl)propanoic acid	0.92 ± 0.34 [a]	3.43 ± 1.88 [a]	0.82 ± 0.38 [a]	8.24 ± 4.38 [a]	42.4 ± 5.99 [b]	5.62 ± 2.50 [a]
3-(3’-hydroxy-4’-methoxyphenyl)propanoic acid	0.68 ± 0.22 [a]	1.34 ± 0.52 [a]	0.67 ± 0.25 [a]	4.37 ± 2.13 [a]	16.5 ± 3.36 [b]	1.87 ± 0.65 [a]
3-(3,5-dimethoxy-4-hydroxyphenyl)propanoic acid	0.21 ± 0.06 [a]	1.77 ± 0.51 [a,b]	0.87 ± 0.35 [a,b]	1.07 ± 0.30 [a,b]	1.87 ± 0.66 [a,b]	2.95 ±1.60 [b]
3-(3,5-dimethoxy-4-hydroxyphenyl)propanoic acid-*O*-sulphate (I)	0.19 ± 0.09 [a]	0.09 ± 0.06 [a]	2.56 ± 1.32 [a]	52.8 ± 32.2 [a]	327 ± 53.3 [b]	52.72 ± 28.7 [a]
3-(3,5-dimethoxy-4-hydroxyphenyl)propanoic acid-*O*-sulphate (II)	4.74 ± 1.93 [a]	9.87 ± 3.42 [a]	4.32 ± 3.24 [a]	17.8 ± 11.0 [a]	87.8 ± 21.4 [b]	17.3 ± 7.40 [a]
**Phenylacetic and phenylpropanoic acids**						
4’-Hydroxyphenylacetic acid sulphate	31.3 ± 4.92 [a]	39.6 ± 16.1 [a]	40.2 ± 11.5 [a]	47.9 ± 14.6 [a]	87.0 ± 38.4 [a,b]	154 ± 77.4 [b]
2’-Hydroxyphenylacetic acid sulphate	17.3 ± 1.68 [a]	6.41 ± 2.90 [a]	12.8 ± 6.09 [a]	17.8 ± 6.81 [a]	67.0 ± 39.9 [a,b]	112 ± 49.3 [b]

Hydroxyphenylacetic acid-*O*-sulphate	1.98 ± 0.39 ^{a}	2.54 ± 0.67 ^{a}	1.86 ± 0.55 ^{a}	1.37 ± 0.68 ^{a}	20.0 ± 6.21 ^{b}	33.4 ± 11.6 ^{b}
Hydroxyphenylacetic acid-*O*-glucuronide	0.00 ± 0.00 ^{a}	0.11 ± 0.06 ^{a}	0.00 ± 0.00 ^{a}	0.03 ± 0.03 ^{a}	1.91 ± 0.44 ^{c}	0.94 ± 0.42 ^{b}
3',4'-Dihydroxyphenylacetic acid-*O*-sulphate	10.3 ± 4.36 ^{a}	11.87 ± 3.93 ^{a}	27.5 ± 6.71 ^{a}	44.9 ± 12.9 ^{a}	98.2 ± 27.13 ^{b}	36.6 ± 11.8 ^{a}
3',4'-Dihydroxyphenylacetic acid-*O*-glucuronide	0.88 ± 0.31 ^{a}	1.45 ± 0.38 ^{a}	3.85 ± 0.93 ^{a,b}	2.93 ± 0.52 ^{a,b}	20.3 ± 12.0 ^{b}	13.4 ± 9.36 ^{a,b}
3-(4'-Hydroxyphenyl)propanoic acid	0.55 ± 0.42 ^{a}	0.52 ± 0.29 ^{a}	0.95 ± 0.72 ^{a}	4.64 ± 2.69 ^{a,b}	20.0 ± 9.23 ^{c}	15.2 ± 6.27 ^{b,c}
3-(4'-Hydroxyphenyl)propanoic acid-*O*-sulphate	21.9 ± 10.5 ^{a}	13.2 ± 5.92 ^{a}	5.87 ± 3.34 ^{a}	17.3 ± 13.4 ^{a}	216 ± 79.2 ^{b}	192 ± 65.5 ^{b}
3-(4'-Hydroxyphenyl)propanoic acid-*O*-glucuronide	0.04 ± 0.04 ^{a}	0.06 ± 0.04 ^{a}	0.08 ± 0.08 ^{a}	0.00 ± 0.00 ^{a}	0.49 ± 0.26 ^{b}	0.00 ± 0.00 ^{a}
3-(3',4'-Dihydroxyphenyl)propanoic acid	1.25 ± 0.77 ^{a}	2.83 ± 1.25 ^{a}	0.95 ± 0.60 ^{a}	4.49 ± 2.61 ^{a}	41.7 ± 23.6 ^{a,b}	98.0 ± 51.1 ^{b}
3-(3',4'-Dihydroxyphenyl)propanoic acid-*O*-sulphate (I)	8.27 ± 3.22 ^{a}	3.71 ± 1.51 ^{a}	5.97 ± 1.97 ^{a}	3.74 ± 1.23 ^{a}	10.2 ± 3.68 ^{a,b}	19.7 ± 7.75 ^{b}
3-(3',4'-Dihydroxyphenyl)propanoic acid-*O*-sulphate (II)	9.41 ± 3.00 ^{a,b}	3.42 ± 1.58 ^{a}	5.06 ± 1.88 ^{a}	16.6 ± 6.14 ^{a,b}	48.9 ± 17.1 ^{c}	34.9 ± 13.3 ^{b,c}
3-(3',4'-Dihydroxyphenyl)propanoic acid-*O*-glucuronide	0.19 ± 0.04 ^{a}	0.08 ± 0.03 ^{a}	0.25 ± 0.11 ^{a}	0.24 ± 0.15 ^{a}	1.41 ± 0.58 ^{a,b}	1.85 ± 1.09 ^{b}
Benzene-1,2-diol derivatives						
1,2-Dihydroxybenzene-*O*-sulphate (I)	55.5 ± 10.7 ^{a}	24.1 ± 9.73 ^{a}	41.2 ± 24.20 ^{a}	110 ± 43.15 ^{a}	1266 ± 250 ^{c}	712 ± 211 ^{b}
1,2-Dihydroxybenzene-*O*-sulphate (II)	15.1 ± 2.97 ^{a}	23.0 ± 7.92 ^{a,b}	10.7 ± 4.41 ^{a}	9.60 ± 5.62 ^{a}	13.80 ± 7.78 ^{a}	48.7 ± 23.6 ^{b}
Methoxy-hydroxybenzene-*O*-sulphate (I)	6.91 ± 1.14 ^{a}	6.13 ± 3.48 ^{a}	7.74 ± 3.73 ^{a}	8.71 ± 4.75 ^{a}	98.4 ± 25.5 ^{c}	50.5 ± 21.58 ^{b}
Methoxy-hydroxybenzene-*O*-sulphate (II)	0.14 ± 0.48 ^{a}	0.80 ± 0.00 ^{a}	n.d. ^{a}	n.d. ^{a}	3.64 ± 3.06 ^{a,b}	9.10 ± 6.33 ^{b}
1,2-Dihydroxybenzene-*O*-glucuronide (I)	0.13 ± 0.02 ^{a}	0.35 ± 0.08 ^{a}	0.37 ± 0.20 ^{a}	0.26 ± 0.13 ^{a}	1.33 ± 0.56 ^{b}	1.36 ± 0.53 ^{b}
1,2-Dihydroxybenzene-*O*-glucuronide (II)	n.d. ^{a}	0.01 ± 0.01 ^{a}	0.03 ± 0.03 ^{a}	0.00 ± 0.00 ^{a}	0.44 ± 0.30 ^{a,b}	1.08 ± 0.54 ^{b}
Phloroglucinol/pyrogallol derivatives						
Benzene-1,2,3-triol-*O*-sulphate or benzene-1,3,5-triol-*O*-sulphate (I)	12.0 ± 2.45 ^{a,b}	1.83 ± 0.79 ^{a}	4.87 ± 2.23 ^{a}	10.7 ± 3.93 ^{a}	88.6 ± 55.8 ^{b}	72.5 ± 35.3 ^{a,b}
Benzene-1,2,3-triol-*O*-sulphate or benzene-1,3,5-triol-*O*-sulphate (II)	53.3 ± 17.3 ^{a}	1.29 ± 1.29 ^{a}	6.85 ± 5.60 ^{a}	60.9 ± 60.51 ^{a,b}	81.2 ± 40.4 ^{a,b}	192.8 ± 90.6 ^{b}
Methoxy-dihydroxybenzene-*O*-sulphate (I)	14.4 ± 4.96 ^{a}	29.5 ± 12.8 ^{a}	29.1 ± 9.94 ^{a}	293 ± 80.3 ^{b}	634 ± 177 ^{c}	68.8 ± 20.0 ^{a,b}
Methoxy-dihydroxybenzene-*O*-sulphate (II)	3.44 ± 1.09 ^{a}	1.31 ± 0.61 ^{a}	1.40 ± 1.03 ^{a}	1.10 ± 1.10 ^{a}	18.2 ± 4.62 ^{b}	21.5 ± 6.47 ^{b}
Methoxy-dihydroxybenzene-*O*-glucuronide	0.18 ± 0.06 ^{a}	0.62 ± 0.30 ^{a}	0.45 ± 0.15 ^{a}	3.77 ± 1.05 b	8.27 ± 2.19 ^{c}	1.69 ± 0.61 ^{a,b}

* Concentration (nmols)
Different letters means statistically different ($p < 0.05$).
n.d.: not detected
