# Supplementary material for: Bioavailability and Metabolic Fate of (Poly)phenols from Hull-Less Purple Whole-Grain Barley in Humans
Source: Nutrients. 2025 Sep 28;17(19):3086. doi: 10.3390/nu17193086 (PMC12526210; doi:10.3390/nu17193086)
Supplement: Supplementary file 1 [file nutrients-17-03086-s001.zip › Supplementary Figure S1_Cortijo-Alfonso_Nutrients.pptx]

## Slide 1
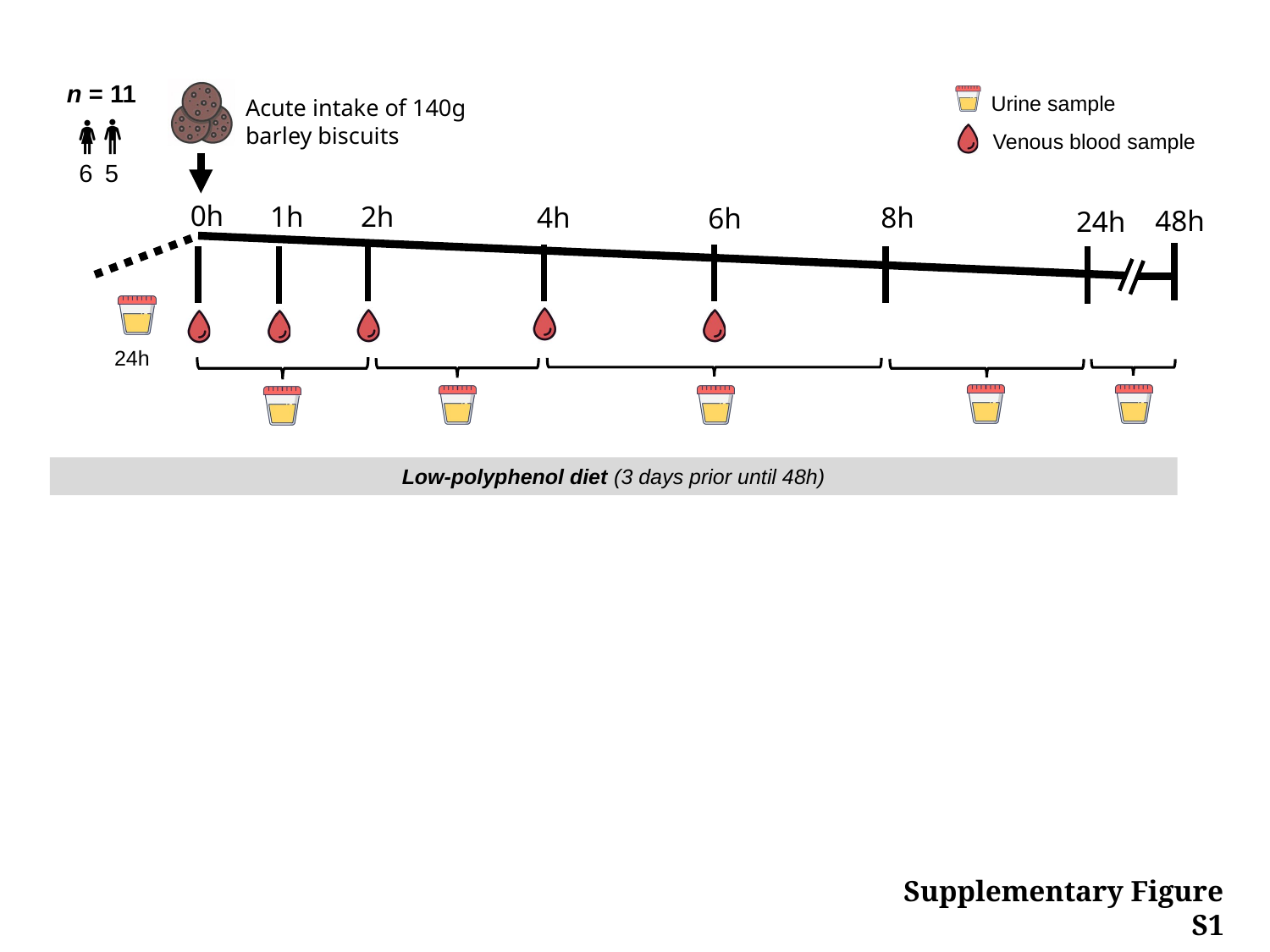

n = 11
Urine sample
Acute intake of 140g barley biscuits
Venous blood sample
6
5
0h
2h
1h
4h
8h
6h
48h
24h
24h
Low-polyphenol diet (3 days prior until 48h)
Supplementary Figure S1
