# Supplementary material for: Bioavailability and Metabolic Fate of (Poly)phenols from Hull-Less Purple Whole-Grain Barley in Humans
Source: Nutrients. 2025 Sep 28;17(19):3086. doi: 10.3390/nu17193086 (PMC12526210; doi:10.3390/nu17193086)
Supplement: Supplementary file 1 [file nutrients-17-03086-s001.zip › Supplementary Figure S3_Cortijo-Alfonso_Nutrients_02.pptx]

## Slide 1
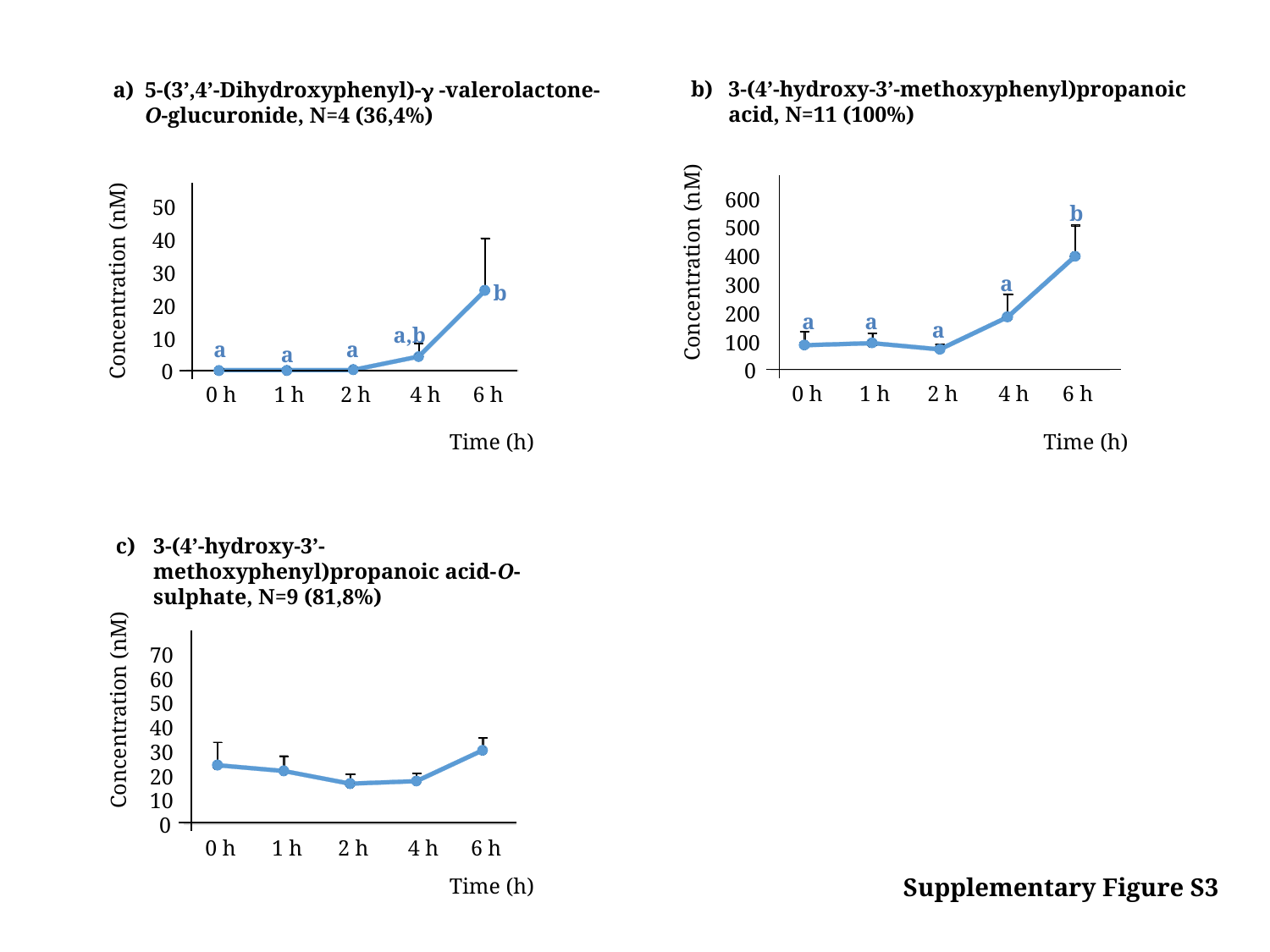

3-(4’-hydroxy-3’-methoxyphenyl)propanoic acid, N=11 (100%)
600
b
500
400
Concentration (nM)
a
300
200
a
a
a
100
0
0 h
1 h
2 h
 4 h
6 h
Time (h)
5-(3’,4’-Dihydroxyphenyl)-γ -valerolactone-O-glucuronide, N=4 (36,4%)
50
40
30
Concentration (nM)
b
20
a,b
10
a
a
a
0
0 h
1 h
2 h
 4 h
6 h
Time (h)
3-(4’-hydroxy-3’-methoxyphenyl)propanoic acid-O-sulphate, N=9 (81,8%)
70
60
50
Concentration (nM)
40
30
20
10
0
0 h
1 h
2 h
 4 h
6 h
Time (h)
Supplementary Figure S3
