# Supplementary material for: Bioavailability and Metabolic Fate of (Poly)phenols from Hull-Less Purple Whole-Grain Barley in Humans
Source: Nutrients. 2025 Sep 28;17(19):3086. doi: 10.3390/nu17193086 (PMC12526210; doi:10.3390/nu17193086)
Supplement: Supplementary file 1 [file nutrients-17-03086-s001.zip › Supplementary Figure S4_Cortijo-Alfonso_Nutrients_02.pptx]

## Slide 1
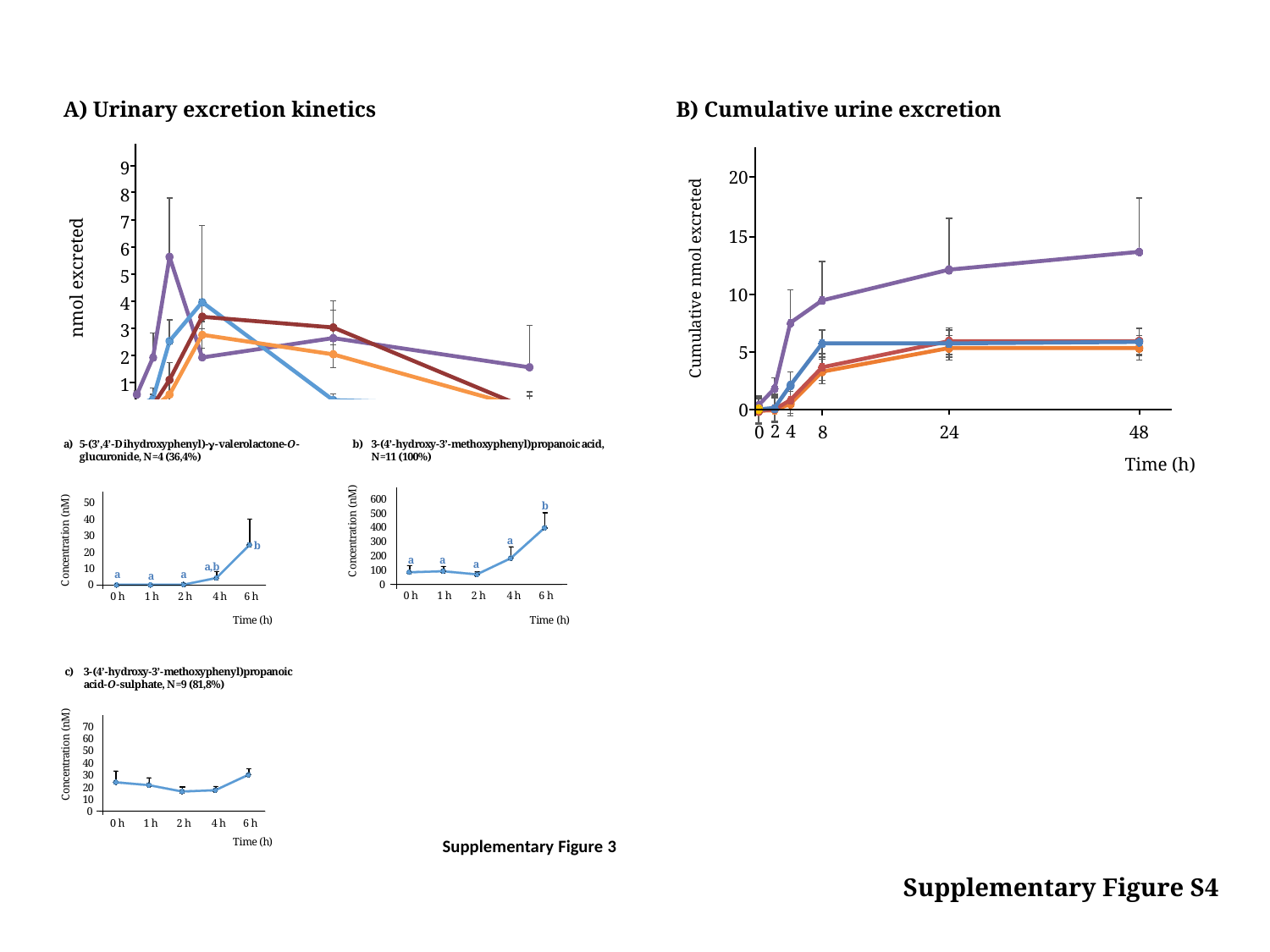

B) Cumulative urine excretion
A) Urinary excretion kinetics
9
20
8
7
15
6
5
nmol excreted
Cumulative nmol excreted
10
4
3
5
2
1
0
0
10
20
30
40
50
2
4
8
0
24
48
Time (h)
Time (h)
Peonidin-3-O-glucoside
Peonidin-3-O-glucuronide
Cyanidin-3-O-(6’’-O-malonyl)glucoside
Cyanidin-3-O-(3’’-O-malonyl)glucoside
Supplementary Figure S4
